# Longitudinal Follow-Up of Clinical Superficial Ovine Caseous Lymphadenitis

**DOI:** 10.3390/ani14243641

**Published:** 2024-12-17

**Authors:** Nora El Khalfaoui, Bouchra El Amiri, Abdellatif Rahim, Mouad Chentouf, Marianne Raes, Tanguy Marcotty, Nathalie Kirschvink

**Affiliations:** 1 Namur Research Institute for Life Sciences NARILIS, University of Namur, 5000 Namur, Belgium; nora.elkhalfaoui@unamur.be (N.E.K.);; 2 Animal Production Unit, Regional Center Agricultural Research of Settat, National Institute for Agricultural Research (INRA), Avenue Ennasr, P.O. Box 415, Rabat Principal, Rabat 10090, Morocco; 3 Laboratory of Biochemistry, Neurosciences, Natural Resources and Environment, Faculty of Sciences and Techniques, Hassan First University of Settat, P.O. Box 577, Settat 26000, Morocco; 4 Regional Center of Agricultural Research of Tangier, National Institute for Agricultural Research (INRA), Avenue Ennasr, P.O. Box 415, Rabat Principal, Rabat 10090, Morocco

**Keywords:** caseous lymphadenitis, clinical incidence, season, relapse, risk factors, Settat province

## Abstract

Caseous lymphadenitis is an infectious disease that has a significant economic impact on sheep breeding. The objectives of this study were to evaluate the effect of season, animals’ age, sex, body score and shearing on the clinical incidence of caseous lymphadenitis, relapses and abscess location in sheep from Settat province, Morocco. In this longitudinal study, 274 clinically healthy sheep were recruited in six flocks among 1451 inspected sheep and followed during 12 months to cover four seasons. The animals involved in this study were assigned to different age categories at continuous recruitment from February to July 2021 (lambs younger than 6 months, young sheep between 6 and 24 months and old sheep more than 24 months) and to sex categories. Selected animals were clinically examined every 2 months to detect superficial abscesses by palpation of superficial lymph nodes. Information about the number of abscesses and their locations was recorded and analyzed in multivariable statistical models. The results showed that 185/274 (67.5%) of the monitored animals developed superficial CL abscesses. The risk ratio (RR) of superficial CL was significantly higher between April and September compared to between January and March (RR~4.4; *p* < 0.0001). No significant difference was found between October and December compared to between January and March (RR = 1.2; *p* = 0.64). Regarding the effect of age, the results revealed that the RR was significantly lower in old sheep compared to lambs (RR = 0.45, *p* < 0.0001). No significant difference was detected between lambs and young sheep (RR = 0.7, *p* = 0.07). The prevalence of caudally located abscesses (prefemoral and popliteal lymph nodes, as well as in testicles, scrotum and mammary glands) was significantly higher in old sheep than in lambs (20% versus 3%; odds ratio = 7.8, *p* = 0.02). The sex, body score and shearing since the last examination did not show any significant effect on CL incidence (*p* > 0.1). Abscess relapse was significantly lower in old sheep than in lambs (IRR = 0.4, *p* = 0.003). The highest clinical CL incidence was observed in young animals between April and September and was likely due to indoor intensive rearing management. To conclude, both season and age had significant effects on superficial CL incidence. Farmers, veterinarians and technicians should focus attention and preventive measures against CL on young animals during spring and summer.

## Simple Summary

Caseous lymphadenitis (CL) is an infectious disease clinically characterized by suppurative necrotic inflammation in superficial and internal lymph nodes and organs. This pathology is responsible for economic impacts in the sheep industry worldwide. In Morocco, where sheep farming is considered an important source of income for the rural population, CL causes significant economic losses to breeders due to the low prices and limited saleability of sheep showing superficial lesions. In this study, a 12-month longitudinal follow-up was carried out to evaluate the effect of seasons, the animals’ age, sex, body score and shearing on the clinical incidence of CL, relapses and abscess location in sheep. As a result, both season and age showed significant effects on superficial CL incidence. A high incidence of CL in lambs and young sheep occurred between April and September, which coincided with the fattening periods and indoor housing. It appears promising to focus attention and preventive measures against CL on young animals during spring and summer to improve CL control strategies.

## 1. Introduction

Caseous lymphadenitis (CL) is an infectious disease caused by *Corynebacterium pseudotuberculosis*, which is responsible for sanitary and economic impacts in sheep breeding areas where the disease is endemic. This condition is known as cutaneous or superficial when it infects superficial lymph nodes, e.g., parotid, retropharyngeal, submandibular, popliteal, prefemoral and prescapular lymph nodes. Another form of CL exists and concerns the visceral or internal organs. It is characterized by the infection of mediastinal and bronchial lymph nodes and viscera, including the lungs, spleen, kidneys and liver [1]. When abscesses are located in retropharyngeal lymph nodes, the animals might suffer from reduced food intake. Important weight loss is attributed to visceral CL forms due to reduced digestive function. Earlier studies reported an association between loss of body weight and *C. pseudotuberculosis* infection. CL was associated with loss of body weight in infected goats [2]. Additionally, a recent study reported a significant body weight loss in an inoculated goat group with *C. pseudotuberculosis* [3]. As economic consequences of this disease, a decrease in meat yield due to contaminated carcasses and trimmed-out portions has been recorded [4]. Additionally, wool production, leather and reproductive efficiency are also negatively impacted by this disease [5,6]. In Morocco, where sheep breeding is one of the most important agricultural activities, CL is responsible for considerable economic losses to sheep breeders [7]. This negative impact is mainly due to the low saleability and prices of sheep presenting external lesions, especially during religious celebrations. Such an effect is remarkably recorded in Sardi sheep, one of the most popular breeds in Morocco, because of its large, uncovered skin zone allowing for easy detection of the external lesions. Furthermore, because of its zoonotic characteristics, CL could infect humans. More precisely, zoonotic CL has been reported among farmers, veterinarians and shear workers who were in close contact with infected animals [8,9,10]. This disease has a cosmopolitan distribution and affects mainly small ruminants. It is also described in other mammalian species such as cattle, horses and camelids [11,12].

In sheep, CL is caused by *Corynebacterium pseudotuberculosis biovar ovis*, a Gram-positive, facultative intracellular bacterium. The first isolation and description of this bacterium was realized in 1888 by Nocard from a case of ulcerative lymphangitis in bovine. It was previously named Preisz-Nocard bacillus, Preisz-Guinard bacillus and *Corynebacterium ovis* [13]. The main important virulence factors contributing to *C. pseudotuberculosis* pathogenicity are the external lipids of the bacterial wall and phospholipase D exotoxin. The former promotes local cytotoxicity by protecting against phagolysosomes’ proteolytic enzymes, allowing the adherence of the bacterium. The phospholipase D exotoxin dissociates sphingomyelin, a component of the double lipid layer of the cell membrane, and vascular endothelium into ceramid phosphate and cholin [14,15,16]. This causes great damage to the host cell membranes with probable dysfunction, making *C. pseudotuberculosis* more resistant to phagocyte destruction [10,17,18]. After a period of incubation, the infection with *C. pseudotuberculosis* leads to pyogranulomatous lesions, then the abscesses become swollen and encased within fibrous capsules [5,10,19]. The incubation period of CL from inoculation to abscessation is variable from 2 weeks to 6 months in sheep and goats [19,20,21]. Furthermore, another study reported CL as a subacute disease in goats with an incubation period of 8–9 days, serologically undetectable till 15 days after the infection [22]. Till now, although there is sensitivity of the bacteria against antimicrobial agents, there is no efficient treatment of CL, and almost all antibiotherapies have failed. This is mainly due to the intracellular character of the bacterium and the fibrous capsule surrounding the lesions [23,24]. Curettage of abscesses is no longer recommended due to the increased risk of germ dissemination [4]. Vaccination is considered an important tool to limit the spread of the disease by preventing the infection of naïve animals [25].

If the bacterium is introduced in a flock, the disease becomes endemic and difficult to eradicate, notably because of its survival ability in the soil, which can reach several months [18]. Al-Rawashdeh and Al-Qudah reported that the prevalence of CL increased with the animals’ age and that the incidence increased in young sheep after shearing [26]. However, very few studies concerned the evolution of CL incidence in function of the animals’ age and sex. Yitagesu et al. reported that CL incidence was significantly higher in kids and females than in yearling and male goats, respectively [27]. Very likely, the incidence of CL in sheep may be variable over time (seasons) depending on the management changes considering the animals’ age. In Settat province, our previous survey was carried out on 1521 sheep belonging to 56 flocks where CL was confirmed clinically and by quadruplex PCR on isolated colonies. A high percentage of superficial CL-form-affected flocks (95%) with a mean clinical prevalence of 34.6% was reported. Age (6 to 24 months) appeared as a risk factor [28]. To investigate the occurrence of CL and its evolution in this province, a longitudinal study was carried out for future improvement of the control strategies against this disease. Therefore, the objectives of this study were (i) to estimate the clinical CL incidence in function of the animals’ age, sex, body score and shearing history, (ii) to determine the risk of abscess relapse in function of the animals’ age and sex, and (iii) to assess whether abscess location is correlated with the animals’ age.

## 2. Materials and Methods

### 2.1. Study Zone

The Beni-Meskine area belongs to the circle of El Brouj (32°30′14.3′′ N–7°11′21.0′′ W) in the Casablanca-Settat region and is located in the central region of Morocco. It covers an area of approximately 200 km^2^, including 11 rural communes at an average altitude of 800 m. The climate of this area is arid with hot summers and cold winters. High daily and seasonal thermal amplitudes are recorded. The lowest mean temperatures are recorded in January (5 °C), and the highest mean temperatures are recorded in August (40 °C). The average annual rainfall is around 270 mm with significant inter- and intra-annual variability. The land is generally rocky. The territory of Beni-Meskine is dominated by limestone formations of Cretaceous and tertiary age, which extend to the south of the phosphate area.

### 2.2. Study Design

**Figure 1:**
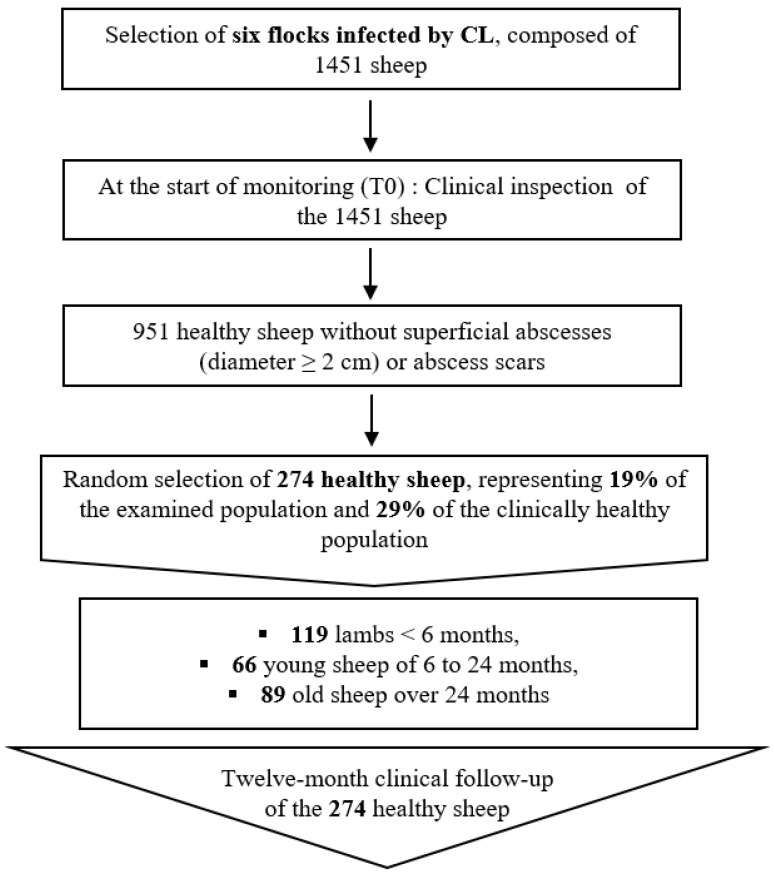
Diagram summarizing the selection steps of the 274 chosen sheep for the follow-up.

**Figure 2:**
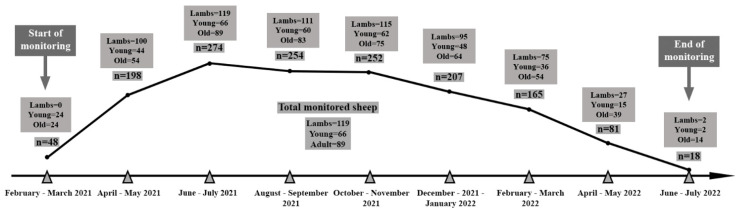
Sheep enrollment at T0 and 12 months follow-up, n = total number of animals examined at each time point. Lambs: sheep < 6 months; young: sheep from 6 to 24 months; old: sheep > 24 months.

Based on flock owner compliance and willingness to enroll sheep in a longitudinal study for twelve months, sheep belonging to six flocks were selected according to the procedure depicted in Figure 1. The herds had never been vaccinated against CL and were previously confirmed as infected by *C. pseudotuberculosis* by means of a quadruplex PCR on suspected isolated and purified colonies [29]. Primers that target the *16S rRNA*, *rpoB* and *pld* genes were used in the quadruplex PCR [30,31,32]. The PCR was realized directly on isolated colonies of *C. pseudotuberculosis* according to the protocol described in our previous study [28]. The enrollment of sheep for monitoring started between February and July 2021 (T0) and ended between February and July 2022 (Figure 2). The enrollment of sheep and particularly lambs occurred gradually because of the non-synchrony of the lambing period. Consequently, the 12-month follow-up of selected sheep started and ended at different time points. We aimed at sampling animals of three age categories that belonged to six flocks. To detect differences between two proportions ranging from 25 to 50% with a power of 90%, confidence of 95% and within-flock correlation of 1%, a total of 285 samples would be required. After an initial screening including all animals of each flock (total number of sheep = 1451), around 30% of clinically healthy lambs and young and old sheep were selected in each flock for the longitudinal follow-up (Supplemental Table S1). Enrollment limitation of young and old sheep was mainly due to flock owners who could not ensure keeping those sheep for 12 months. The enrolled farms were visited every two months, and selected animals underwent clinical examination.

Three age categories of animals were established on all farms: lambs (aged less than 6 months), young sheep (aged from 6 to 24 months) and old sheep (more than 24 months). The rearing systems were similar in four farms and a little different in the other two farms; see Table S1 of the Supplementary Material. Young animals were raised indoors between 6 and 24 months of age on farms 1, 3, 4 and 5. In farm 2, all animals were reared semi-extensively. In farm 6, young animals were under indoor intensive management between May and July. Indoors, animals were fed with alfalfa hay supplemented with barley grains and bran. Sheep raised in a semi-extensive rearing system grazed outdoors for most of the day and stayed indoors during the night, whereas those raised under the intensive system were kept indoors day and night. Consequently, the rearing characteristics cited below were used for analysis.

In addition to age and sex, body score and shearing were recorded for each enrolled animal. The body score of each recruited animal was recorded at the beginning of this study and then at every observation during the follow-up using a score from 1 to 5 according to a detailed technical sheet of the national agricultural council office [33]. Initial body condition score was distributed as follows: among all selected sheep, score 2 was predominantly recorded (43%), followed by score 3 (27%), score 1 (24%) and rarely score 4 (6%), while score 5 was not observed. By considering age categories, 60% of lambs had score 2, followed by 25% with score 3; 52% of young sheep had score 3, followed by 21% with score 4; and 45% of old sheep had score 1, followed by 42% with score 2. Information about shearing since the last examination was recorded only during the post-shearing period, from April to May. A total of 128 females and 18 males, including 19 lambs, 43 young sheep and 84 old sheep, were sheared since the preceding examination and examined during the post-shearing period.

In order to analyze the impact of the season of the calendar year, four seasons were defined: season 1 (January–February–March), corresponding to the middle of the lambing period, characterized by maximal and minimal mean temperatures of 20 °C and 9 °C, respectively; season 2 (April–May–June), corresponding to the late lambing period and beginning of the fattening period, characterized by maximal and minimal mean temperatures of 30 °C and 14 °C, respectively; season 3 (July–August–September), corresponding to the fattening period, characterized by maximal and minimal mean temperatures of 35 °C and 20 °C, respectively; and season 4 (October–November–December), corresponding to the beginning and peak of the lambing period, characterized by maximal and minimal mean temperatures of 26 °C and 16 °C, respectively (Figure S1).

### 2.3. Clinical Examination

At T0, all animals of each flock underwent clinical examination, including determination of age (lamb < 6 months, young: 6–24 months, old sheep > 24 months), sex, body condition score and presence of superficial abscesses. Parotid, submandibular, retropharyngeal, prescapular, prefemoral and popliteal lymph nodes, as well as testicles and scrotum in rams and mammary glands in ewes, were carefully palpated. The presence of one or more palpable abscesses (diameter ≥ 2 cm) or abscess scars, as well as its/their location, was recorded. Sheep without abscesses or abscess scars were eligible for follow-up and were identified by ear tags. During the follow-up, the first occurrence of abscesses and recurrent ones was recorded. The age of animals at enrollment was estimated by incisor teeth examination, considering the replacement of temporary incisors by permanent ones as previously described by El Khalil et al. [34]. For animals with temporary teeth, the information about the age was given directly by the farmers. Each enrolled sheep underwent clinical examination every two months, including abscess or abscess scar detection, determination of body score and recording of shearing information since the last examination (sheared or not sheared). Abscess location was systematically recorded. Regarding age category, each animal was considered as belonging to its initial age category. All movements of the animals (sale, death or transfer to unavailable locations) were recorded.

### 2.4. Statistical Analysis

All analyses were performed in Stata (StataCorp, version 11, College Station, TX, USA).

#### 2.4.1. Survival Analysis of CL Incidence Risk

The survival rate was considered as the proportion of healthy animals showing no superficial abscesses during the period of the clinical follow-up. Survival analysis models were developed using single failure data (animals are removed from the cohort when found positive for CL). First, a semi-parametric Cox model was applied to evaluate the variability of CL incidence across flocks (“flocks” was used as a single explanatory variable). Frailty exponential models were subsequently designed using “flocks” as a shared frailty variable and sex, age categories, body score, shearing and seasons as discrete explanatory variables. The significance of explanatory variables was evaluated in univariable and multivariable models. For all multivariable models, two-by-two interactions were tested with all significant explanatory variables (*p* < 0.05). Insignificant interactions (*p* > 0.05) were ignored. By considering the first detection of an abscess as a failure event, Kaplan–Meyer curves were created for variables of interest (season, sex and age categories). A final multivariable predictive model was developed using only significant explanatory variables (*p* < 0.05).

#### 2.4.2. Age and Abscess Locations

Abscess location, considering age categories, was analyzed using robust logistic regressions by pooling abscesses detected in parotid, submandibular, retropharyngeal and prescapular lymph nodes (i.e., proximal location) and grouping abscesses in prefemoral and popliteal lymph nodes as well as in testicles, scrotum and mammary glands (i.e., caudal location). A database subset of all abscesses observed (including relapses) was used, and whether an abscess was proximal or not was the binary response. Sex, age category and season were evaluated as explanatory variables in a multivariable model.

#### 2.4.3. Abscess Relapses

The occurrence of abscess relapses was evaluated in robust multivariable Poisson models (number of CL episodes observed in one animal during the period of observation), using flocks as clusters and age and sex categories as explanatory variables. The response was the total number of abscesses recorded in each animal during the follow-up period. The number of examinations that individual animals underwent was used as exposure in the Poisson regression. Incidence rate ratio (IRR) was compared between categories of each explanatory variable.

## 3. Results

During the follow-up, a total of 334 abscesses were detected in 185 animals. Among those, 49% displayed a new abscess at least once. Table 1 displays the number of animals with different numbers of CL episodes in different age groups.

### 3.1. Incidence of Superficial Caseous Lymphadenitis

The distribution of clinical observations, clinical detection of CL and results of univariable survival analysis by flock, season, age category, sex, body score and shearing information since the last examination are detailed in Table 2. The semi-parametric Cox model revealed significant variations in the risk of developing CL abscesses across flocks. Both univariable and multivariable survival analyses showed significant effects of seasons and age categories (*p* < 0.001). In the univariable survival analysis, it was revealed that the males were non-significantly less infected by superficial CL (OR = 0.89, *p* = 0.52), the body score non-significantly increased with the superficial CL incidence contrasting our hypothesis, whereas the shearing since the last examination had no negative effect on its incidence. No significant two-by-two interaction between season, age and sex was observed (*p* > 0.1). Therefore, subsequent analysis focusing on the effects of seasons, age and sex was undertaken.

**Figure 3:**
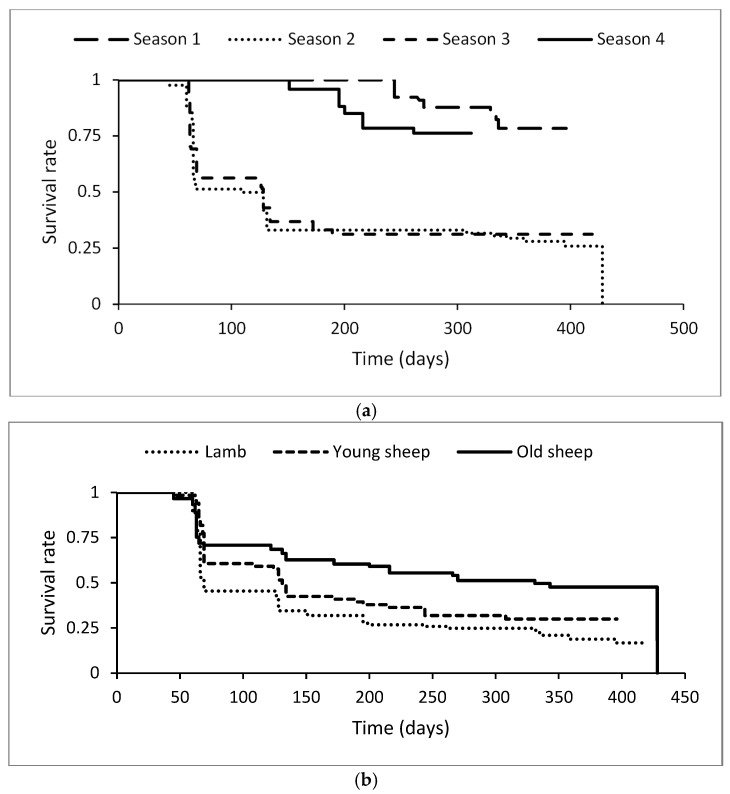
Kaplan–Meier survival curves of new clinical CL cases from T0 to the end of follow-up, by season (**a**) and age category (**b**): season 1: January–February–March; season 2: April–May–June; season 3: July–August–September; and season 4: October–November–December. Lambs: <6 months; young sheep: 6 to 24 months; old sheep: >24 months. The final survival drop is due to the failure of a single old female in season 2.

Figure 3 illustrates the evolution of the healthy animal proportions, showing no superficial abscesses during the period of the clinical follow-up (survival rate in univariable models) according to the different seasons, age categories and sex. The risk ratios indicated below were calculated according to a multivariable model. Concerning the season, the clinical CL survival rate was lower during season 2 and season 3 while it was higher during season 1 and season 4 (Figure 3a). The hazard of superficial CL was 3.7 times higher during season 2 than during season 1 (95% CI = 1.9–7.3, *p* < 0.0001). Also, during season 3, the risk of superficial CL was 4.6 times higher than during season 1 (95% CI = 2.4–8.9, *p* < 0.0001). No significant difference in the hazard risk of superficial CL was found in season 4 when compared to season 1 (RR = 1.2, 95% CI = 0.5–2.6, *p* = 0.64). Regarding the animals’ age, lambs showed the lowest survival rate while the old sheep showed the highest rate (Figure 3b). The risk ratio of superficial CL was significantly lower in old sheep (RR = 0.4, 95% CI = 0.3–0.67, *p* < 0.0001), while no significant difference was shown with young sheep (RR = 0.7, 95% CI = 0.5–1.03, *p* = 0.07). Chi-square test comparison between ratios of young and old sheep showed a significant difference (*p* = 0.02).

**Figure 4:**
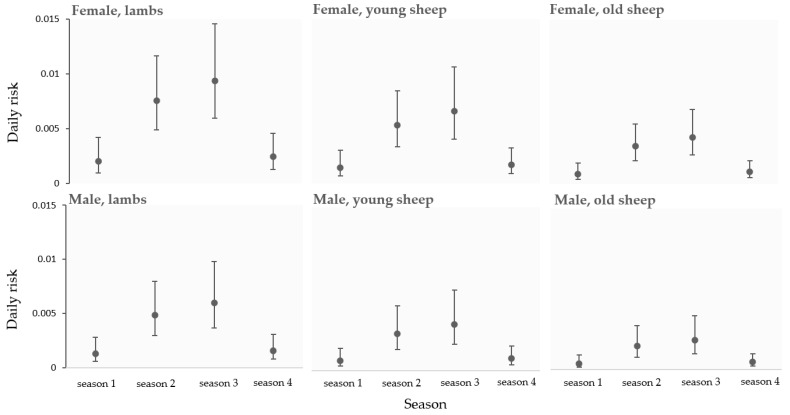
Predictive clinical CL daily risk in function of season and age category in both sexes. Error bars indicate 95% confidence intervals. Interactions were ignored in this predictive model since they were not significant (*p* > 0.05). Lambs: <6 months; young sheep: 6 to 24 months; old sheep: >24 months.

The predictive multivariable model of clinical CL daily hazard in function of seasons and the animals’ age in males and females is detailed in Figure 4. The daily risk of superficial CL was higher in both females and males of all age categories during season 2 and season 3. Moreover, the daily risk of superficial CL decreased with age. It was higher in lambs than in young and old sheep, and it was higher in young than in old sheep, whatever their sex.

### 3.2. Effect of Age on Abscess Locations

Table 3 summarizes the results about abscess locations according to age categories. Whatever the age category, the proximal lymph nodes were the most affected, notably the parotid (50.2%) and prescapular (23.4%) ones.

In a robust multivariable logistic regression, the abscess prevalences of distal locations were significantly higher in old sheep (20%) than in lambs (3%; OR = 7.8, 95% CI = 1.5–40.9, *p* = 0.02). However, it did not differ significantly between lambs and young sheep (8.6%; OR = 2.9, 95% CI = 0.8–11.07, *p* = 0.09). No significant difference was found between young and old sheep categories in the adjusted Wald test (*p* = 0.3).

### 3.3. Abscess Relapse Considering Age and Sex

**Figure 5:**
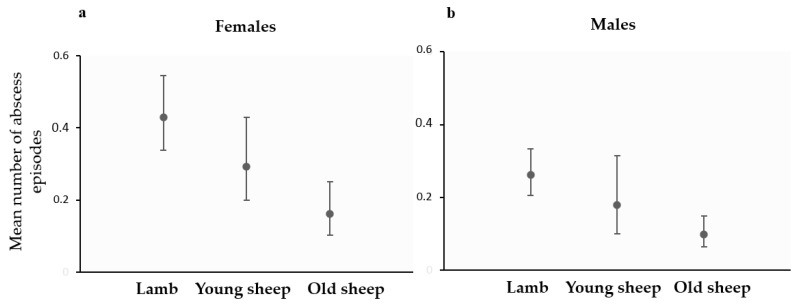
Mean number of abscess episodes detected in female (**a**) and male (**b**) sheep in function of age (lamb: <6 months, young sheep: 6 to 24 months, old sheep: >24 months). Error bars indicate 95% confidence intervals. Interactions were ignored in this predictive model since they were not significant (*p* > 0.05).

In a robust multivariable Poisson regression, abscess relapse was significantly lower in old sheep than in lambs (IRR = 0.4, 95% CI = 0.2–0.6, *p* = 0.003), while it did not differ between lambs and young sheep (IRR = 0.7, 95% CI = 0.4–1.3, *p* = 0.173). No significant difference was found between young and old sheep categories according to the adjusted Wald comparison (*p* = 0.05). Regarding sex, relapse of abscesses was significantly lower in males than in females (IRR = 0.6, 95% CI = 0.4–0.9, *p* = 0.02). The mean abscess incidence in function of age and sex is shown in Figure 5a for females and in Figure 5b for males.

## 4. Discussion

The present study provides current understanding of CL occurrence and relapse in function of seasons, animals’ age, sex, body score and shearing. Our findings revealed that the seasons and the animals’ age impacted CL incidence while sex, body score and shearing were observed to play minor roles. Relapse rate was found to be high, being, however, less important in males and old sheep.

In the present study, a high proportion of new cases occurred during the period of the clinical longitudinal follow-up. The hazard risk of CL varied between flocks. This variability could bias statistical analyses, and therefore, flocks were considered as cluster variables to improve the robustness of subsequent statistical models. It has to be underlined that the lowest hazard risk of CL observed in flock 2 was probably due to its different rearing approach, mainly based on a semi-extensive rearing management for all sheep and the systematic sale of many young animals. This age category showed the highest prevalence of superficial CL in numerous studies, and their systematic sale could decrease the incidence of CL in the flock [17,28,35]. In addition, it was reported in a previous study that the semi-extensive lifestyle reduced the odds ratio of CL [28]. Differences among rearing systems appear as a weakness in our study, where the number of investigated flocks was limited by flock owner compliance and the availability of experienced investigators.

Considering that seasonal reproduction and punctual requests for religious purposes lead to consistent changes in flock size, CL abscess incidence was expected to change over time. Indeed, we observed a higher risk between April and September, mainly related to the indoor intensive management system. The latter is adopted by many farmers, following a genetic scheme selection of the Sardi breed, who are obliged to keep animals of 6 to 18 months indoors to prevent access to collective rangelands highly contaminated with fluorine. Indeed, the Sardi breed is highly susceptible and the most affected by fluorosis among Moroccan sheep breeds [36,37]. Furthermore, in the present study, barns in enrolled flocks were characterized by poor ventilation. The latter was demonstrated as one of the main risk factors implicated in CL occurrence in a previous prevalence study carried out in the same area [28]. Additionally, another explanation for the high superficial CL risk between April and September, especially in lambs and young sheep, is the fact that these categories of sheep are under a fattening process and kept indoors. More precisely, the young animals of 8 to 9 months undergo a 3-month fattening period before the feast of “Aid Al Adha”, which coincided, in the present study, with the months of May, June and July. It should be emphasized that this period changes from one year to the next one with a difference of less than a week since its date follows the Hijri calendar. Regarding old sheep, despite the lowest risk, the predictive analysis revealed that the seasons between April and September were still at higher risk than the seasons from October to March. This higher risk could be due to the contact between ewes and lambs, leading to an increased infection spread. In fact, the lambing period of Sardi sheep is spread from October to July with a peak from October to December. Mixing animals of different age categories may be one of the reasons for the high CL risk in old sheep on some farms.

Independent of the season effect, lambs and young sheep were at a higher risk of CL than old sheep. This result can be explained by the fact that lambs under the age of 6 months mostly undergo primo-infections with an unprompted immunity. Such a result has been reported by Al-Gaabary et al. and Oreiby in young sheep aged between 12 and 24 months [17,35]. Furthermore, in Tunisia, Saïd et al. recorded a higher prevalence of ovine superficial CL in young animals aged 1–2 years with a rate of 10.33% than in 2–5-year-old sheep with a rate of 6.42% and in animals over 5 years old with a rate of 8.86% [38]. In Ethiopia, the hazard risk of CL prevalence was significantly higher in the newborn than in the yearling age group [27].

Moreover, the findings of the current study showed significantly low CL risk in old sheep, which could be due to the semi-extensive rearing management of this age group. In the studied flocks, ewes went to extensive pastures over the day and were kept in sheepfolds overnight. Sheepfolds in these flocks were characterized by an open, unroofed, wide space allowing for continuous exposure to the sun’s heat. In fact, it has been reported that *C. pseudotuberculosis* has a lower survival rate when high temperatures of 42 °C or more are reached [39,40]. Additionally, the treatment of utensils used for shearing with high temperatures reaching 40 °C or more reduced bacterial growth [39]. Furthermore, the presence of thorny plants in extensive pastures could be a source of injuries and skin irritations [7]. However, especially during warm periods, exposure to the sun’s heat and the absence of moist or slurry outdoor conditions promoting bacterial survival could be another explanation for the decreased incidence of CL in old sheep [41]. The dissemination of CL from affected to healthy sheep during the warm period could be due to contact of injured skin with pus from spontaneously ruptured abscesses, especially during indoor housing associated with a high density of animals.

Regarding gender, there was no significant effect on CL incidence. A similar result was found in a previous cross-sectional analysis realized in the same area [28]. In contrast, Yitagesu et al. showed a significantly high risk of infection in female goats compared to bucks in a longitudinal study [27]. Additionally, a significantly high clinical CL prevalence was further reported in ewes: 20.9% versus 15.9% in rams [35]. Such discrepancies could be due to the reduced proportion of adult males in sheep and goat flocks.

In our investigation, the superficial form of CL did not influence the body score (Table 2). This result is in line with a previous transversal study performed in the same area and evidencing the effect of visceral CL body condition [28].

In the present study, no significant variation was found in superficial CL hazard ratios of sheared animals examined after the period of shearing and unsheared animals. The discordance with other results cited below may be due to the low number of observations of sheared animals (n = 146). However, among all cited factors impacting CL, shearing has been reported as one of the main causes of dissemination between sheep in a flock. When the shearing procedure is realized under unhygienic conditions, including the use of contaminated equipment like pus-contaminated shears, the newly shorn and abraded skins provide an infection route for the bacterium *C. pseudotuberculosis* [9,26]. In addition, it was reported that the prevalence of CL increased with age, and its incidence increased only in young sheep aged 1–2 years old after shearing. This finding was explained mainly by the non-application of sanitary precautions such as disinfection of shearing instruments and using a special space for shearing [26]. Thus, further studies are needed to evaluate the shearing impact on CL incidence in the studied area.

Regarding the anatomical location of abscesses, the infection of caudal lymph nodes was more common in old sheep than in lambs and young sheep. This observation could be due to the transmission of the infection from lambs with cranially located abscesses to old ewes during the lactation period. Because one single old male was found infected, it was not possible to statistically evaluate this hypothesis using our data.

The analysis of CL relapses included all data, comprising recurring abscesses, while all analyses above were performed using only the first CL occurrence. Coming back to the relapsing infections, the occurrence of superficial CL was reported throughout all seasons, and approximately half of total abscess cases corresponded to recurrent CL. Our findings revealed that the relapsing infection was less recorded in males and old sheep. The spread of the infection is related to the transmission between animals by contact with the bacterium from ruptured abscesses of other infected animals [25]. In addition, the infected animals could be reinfected by their own ruptured abscesses, especially those located in the head region. Furthermore, the failure of the animals’ immune response to totally eliminate the infection could be mentioned as further reason for recurrent abscesses, especially in young sheep [5]. We hypothesize that the practices adopted in farming sheep are the main reason for the large infection spread. In fact, mature abscesses open spontaneously and are usually not drained or disinfected. As a result, the abundance of infectious material facilitates the spread of the infection in the flock by contamination of the troughs, feeders, soil and walls in barns. Moreover, the bacterium *C. pseudotuberculosis* may survive for several weeks in soil, especially in the presence of organic material. Precisely, it was reported that *C. pseudotuberculosis* could survive for up to 17 weeks in cool, sheltered sites in the shearing shed environment and for 8 months in different types of soil [41]. More recent studies showed that the *C. pseudotuberculosis* biovar Ovis could survive for long periods (80 to 210 days) in soils in the presence of organic material [40,42]. To eradicate this disease, many studies recommend culling or removal of the animals with recurrent abscesses from the flocks [27,43,44]. However, none of these practices had been adopted for the investigated flocks.

## 5. Conclusions

The present study highlighted the higher incidence of clinical superficial CL in lambs and young sheep, with increased infection rates occurring between April and September, coinciding with the fattening periods and indoor housing. The intensive rearing management seems to be the main factor in the spread of CL infections among young animals. To improve CL control, particular attention should be paid to rearing condition improvement and hygiene management, especially for young sheep kept indoors.

## Figures and Tables

**Table 1:** Number of abscess episodes * in monitored animals in function of age category (non-cumulative figures).

Age Category	No Episode	1 Episode	2 Episodes	3 Episodes	4 Episodes or More	Total
Lambs	25	37	28	22	7	119
Young sheep	20	25	11	8	2	66
Old sheep	44	32	8	4	1	89
Total	89	94	47	34	10	274

* Abscess episode = presence of one or more abscesses in an animal at a clinical examination session.

**Table 2:** Univariable survival analyses of caseous lymphadenitis in sheep using flock, sex, age category, body condition score, shearing and season as explanatory variables.

Explanatory Variables	Number of Clinical Observations (Total)	Number of New Clinical CL Cases	OR (95% IC)
**Flock** (Cox semi-parametric model)	***p* < 0.001**		
Flock 1	317	55	1
Flock 2	162	11	0.34 (0.2–0.6)
Flock 3	133	26	2.4 (1.5–3.97)
Flock 4	229	47	0.84 (0.6–1.2)
Flock 5	140	16	0.57 (0.3–1.6)
Flock 6	234	30	1.01 (0.6–1.6)
**Seasons** (frailty exponential model)	***p* < 0.001**		
January–February–March (season 1)	242	10	1
April–May–June (season 2)	329	81	4.0 (2.05–7.8)
July–August–September (season 3)	295	78	4.8 (2.5–9.3)
October–November–December (season 4)	349	16	1.3 (0.5–2.8)
**Age categories** (frailty exponential model)	***p* < 0.001**		
Lambs (<6 month)	524	94	1
Young sheep (6–24 months)	290	42	0.70 (0.5–1.03)
Old sheep (>24 months)	401	49	0.50 (0.3–0.7)
**Sex** (frailty exponential model)	***p* = 0.52**		
Male	277	42	0.89 (0.6–1.3)
Female	938	143	1
**Body score (min = 1, max= 5)** (frailty exponential model)	***p* = 0.50**		
Score 1	208	22	1
Score 2	468	78	1.3 (0.8–2.1)
Score 3	463	71	1.4 (0.8–2.5)
Score 4 (3 observations of score 5 are included)	76	14	1.7 (0.8–3.5)
**Sheared since last examination** (frailty exponential model)	***p* = 0.93**		
No	1069	166	1
Yes	146	19	0.97 (0.45–2.04)
**Total of observations**	**1215**	**185**	

**Table 3:** Abscess abundance in function of anatomical location and age category.

Abscess Location	Total (n/%)	Lambs ^1^ (n/%)	Young Sheep ^2^ (n/%)	Old Sheep ^3^ (n/%)
Parotid LN ^4^	170/50.2	110/57.0	31/38.3	29/44.6
Submandibular LN	22/6.5	12/6.2	7/8.6	3/4.6
Retropharyngeal LN	42/12.5	30/15.5	8/9.9	4/6.2
Prescapular LN	79/23.4	35/18.1	28/34.6	16/24.6
Subtotal	313/92.3	187/96.9	74/91.3	52/80.0
Prefemoral LN	21/6.2	4/2.1	5/6.2	12/18.5
Popliteal LN	2/0.6	0	2/2.4	0
Testicular gland	2/0.6	2/1.1	0	0
Mammary gland	1/0.3	0	0	1/1.5
Subtotal	26/7.7	6/3.1	7/8.6	13/20.0
Total	339/100	193/100	81/100	65/100

^1^ <6 months; ^2^ from 6 to 24 months; ^3^ > 24 months. ^4^ Lymph nodes: proximal lymph nodes (parotid, submandibular, retropharyngeal and prescapular) and distal lymph nodes (prefemoral, popliteal, testicular gland and mammary gland).

## Data Availability

The original contributions presented in this study are included in this article; further inquiries can be directed to the corresponding author.

## Supplementary Materials

The following supporting information can be downloaded at: https://www.mdpi.com/article/10.3390/ani14243641/s1, Table S1: Characterization of investigated sheep flocks; prevalence of clinically detectable caseous lymphadenitis and enrolled animals for follow-up at this study’s beginning. Figure S1: Maximal and minimal mean temperatures characterizing the four studied periods.
