# Supplementary material for: Longitudinal Follow-Up of Clinical Superficial Ovine Caseous Lymphadenitis
Source: Animals (Basel). 2024 Dec 17;14(24):3641. doi: 10.3390/ani14243641 (PMC11672427; doi:10.3390/ani14243641)
Supplement: Supplementary file 1 [file animals-14-03641-s001.zip › animals-3227138-supplementary.pdf]

Table S1. Characterisation of investigated sheep flocks, prevalence of clinically detectable caseous lymphadenitis and enrolled animals for follow-up at study begin

Flock N°	Age category	Rearing management	N. animals (a)	N. animals with CL at recruitment		N. animals free of CL at recruitment		N. CL free animals recruited		
				**N (b)**	**% (b/a)**	**N (c)**	**% (c/a)**	**N (d)**	**% (d/a)**	**% (d/c)**
**Flock 1**	Lambs[1]	Semi extensive	75	11	14.7	64	85.3	54	72.0	84.4
	Young sheep[2]	Intensive	240	184	76.7	56	23.3	5	2.1	8.9
	Old sheep[3]	Semi extensive	130	45	34.6	85	65.4	14	10.8	16.5
Total			445	240	53.9	205	46.1	73	16.4	35.6
**Flock 2**	Lambs	Semi extensive	25	0	0.0	25	100.0	4	16.0	16.0
	Young sheep	Semi extensive	18	4	22.2	14	77.8	12	66.7	85.7
	Old sheep	Semi extensive	28	3	10.7	25	89.3	15	53.6	60.0
Total			71	7	9.9	64	90.1	31	43.7	48.4
**Flock 3**	Lamb	Semi extensive	38	9	23.7	29	76.3	13	34.2	44.8
	Young sheep	Intensive	54	25	46.3	29	53.7	11	20.4	37.9
	Old sheep	Semi extensive	50	15	30.0	35	70.0	5	10.0	14.3
Total			142	49	34.5	93	65.5	29	20.4	31.2
**Flock 4**	Lambs	Semi extensive	120	29	24.2	91	75.8	16	13.3	17.6
	Young sheep	Intensive	140	62	44.3	78	55.7	29	20.7	37.2
	Old sheep	Semi extensive	120	23	19.2	97	80.8	19	15.8	19.6
Total			380	114	30.0	266	70.0	64	16.8	24.1
**Flock 5**	Lambs	Semi extensive	29	3	10.3	26	89.7	10	34.5	38.5
	Young sheep	Intensive	50	25	50.0	25	50.0	6	12.0	24.0
	Old sheep	Semi extensive	39	8	20.5	31	79.5	17	43.6	54.8
Total			118	36	30.5	82	69.5	33	28.0	40.2
**Flock 6**	Lambs	Semi extensive	105	4	3.8	101	96.2	22	21.0	21.8
	Young sheep	Semi extensive/intensive (May-July)	80	34	42.5	46	57.5	3	3.8	6.5
	Old sheep	Semi extensive	110	16	14.5	94	85.5	19	17.3	20.2
Total			295	54	18.3	241	81.7	44	14.9	18.3
**Total**	Lambs	-	392	56	14.3	336	85.7	119	30.4	35.4
	Young sheep	-	582	334	57.4	248	42.6	66	11.3	26.6
	Old sheep	-	477	110	23.1	367	76.9	89	18.7	24.3
	Total	-	1451	500	34.5	951	65.5	274	18.9	28.8

[1] <6 months, [2] from 6 to 24 months, [3] > 24 months.

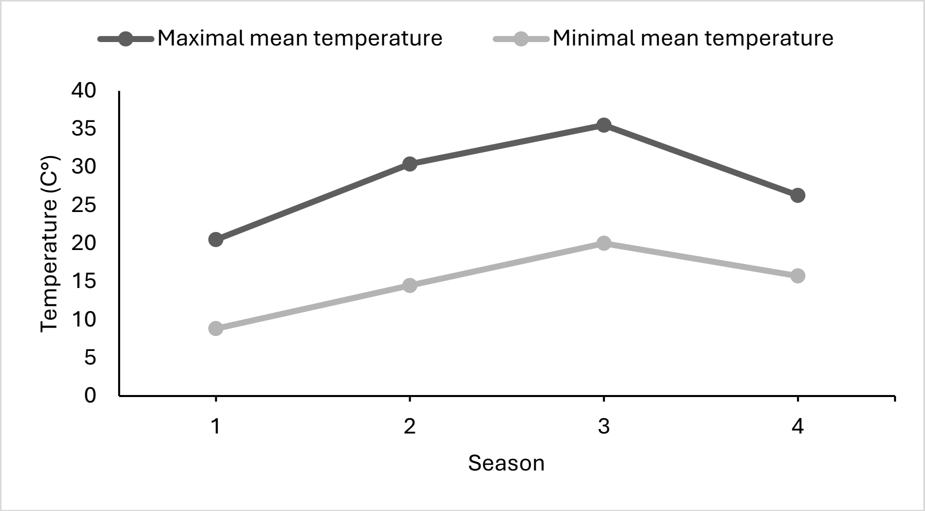

Figure S1. Maximal and minimal mean temperatures characterizing the four studied periods. Season 1: January-February-March, season 2: April-May-June, season 3: July-August-September, season 4: October-November-December. Data collected from: https://www.historique-meteo.net/afrique/maroc/settat/2021/
